# Spectral Exploration of Calcium Accumulation in Organic Matter in Gray Desert Soil from Northwest China

**DOI:** 10.1371/journal.pone.0145054

**Published:** 2016-01-11

**Authors:** Ping Wang, Yucui Ma, Xihe Wang, Hong Jiang, Hua Liu, Wei Ran, Qirong Shen

**Affiliations:** 1 Jiangsu Collaborative Innovation Center for Solid Organic Waste Utilization, Nanjing Agricultural University, Nanjing 210095, China; 2 Research Institute of Soil & Fertilizer and Agricultural Water Conservation, Xinjiang Academy of Agricultural Sciences, Urumqi 830091, China; São Paulo State University, BRAZIL

## Abstract

Little attention has been paid to the accumulation of soil organic matter (SOM) in the fringes of the mid-latitude desert. In this paper, soil samples from a long-term field experiment conducted from 1990 to 2013 at a research station in Urumqi, China by different fertilizer treatments, were used to determine soil properties and soil dissolved organic matter (DOM) by chemical analysis, fluorescence excitation emission matrix (EEM) spectroscopy, and high resolution-transmission electron microscopy (HR-TEM). The binding features of DOM under the addition of Ca^2+^ were analyzed using a two-dimensional (2D) Fourier transform infrared (FTIR) spectrometer further to explore the response of the DOM to increasing concentrations of Ca^2+^. Long-term application of chemical fertilizers and goat manure increased soil organic carbon (SOC) by 1.34- and 1.86-fold, respectively, relative to the non-fertilized control (8.95g.kg^-1^). Compared with the control, application of chemical fertilizers and manure significantly increased the concentrations of Ca, Mg, Si, humic and fulvic acid-like substances in DOM but decreased the amounts of trivalent metals (Al and Fe) and protein-like substances. Although crystalline Al/Fe nanoparticles and amorphous or short-range-order Si/Al nanoparticles existed in all DOM samples, crystalline Ca/Si nanoparticles were predominant in the samples treated with goat manure. Although organic matter and Si-O-containing nanoparticles were involved in the binding of Ca^2+^ to DOM, application of chemical fertilizers weakened Ca^2+^ association with components of the amide II group (1510 cm^-1^) and Si-O linkage (1080 cm^-1^), whereas application of goat manure enhanced the affinity of Ca^2+^ for Si-O linkage. Our results suggested that the enrichment of Ca in gray desert soil possibly helps accumulate SOM by forming crystalline Ca/Si nanoparticles in addition to Ca^2+^ and organic matter complexes.

## Introduction

It is well recognized that SOM is the key component in sustaining soil fertility and conserving environmental quality [1, 2]. Intensive research aimed at enhancing SOM contents or soil carbon storage have been conducted in previous decades[3–5]. Associations of SOM with soil minerals, soil oxides, and soil aggregates have been determined as the main mechanisms by which SOM is protected from microbial decomposition in most soils of the world [6–8]. However, little attention has been paid to SOM dynamic changes in arid desert soils, which contain high amounts of calcium (Ca). The role of Ca in protection of SOM is poorly understood.

Gray desert soil, or haplic calcisol according to the FAO Soil Classification, is a worldwide zonal soil type in mid-latitude temperate deserts or desert steppe climates with approximately 100 to 200 mm of annual rainfall and 1600 to 2100 mm of annual evaporation. The soil originates from loess parent materials and typically contains approximately 50 to 200 g carbonate calcium per kilogram of soil in whole soil profiles with soil pH values ranging from 8.4 to 9.5 [9]. In nature, calcareous soil is dominated by xericshrubs and few of grasses with approximately 10% vegetation cover rates and less than 1% soil organic matter. Previous studies showed that the soil water content profile was determined by the soil texture and root distribution, and it was higher in a clay loam site than that in a sandy loam site[10]. Due to the soil texture, Gray desert suffered from the desertification and salinization. Therefore, the gray desert soil plays an important ecological role as a cultivable agricultural land resource. More than 45000 km^2^ of gray desert soil is distributed in northwestern China [9]. Most of this soil is used for livestock farming, whereas some is converted to agricultural land for vegetable and cotton production. However, the gray desert soil is vulnerable to degradation due to wind erosion, desertification and salinization.

Interestingly, results from a long-term field experiment conducted since 1990 of the effects of agricultural fertilization of wheat and corn on gray desert soil in Urumqi, Xinjiang Province, China, showed that continual application of animal manure plus chemical fertilizer increases soil organic carbon (SOC) up to 23.86 g.kg^-1^ in 2011, which was 2.9- and 2.5-times higher than in the no-fertilizer control (8.33g.kg^-1^) and the chemical fertilizer treatment group (9.41g.kg^-1^), respectively [11]. The results indicate that significant increases in SOM in the gray desert soil around the fringes of desert could sequester much more carbon in the soil and supply more agricultural opportunity than expected. In contrast, few efforts have been made to understand the accumulation of SOM in the soils.

A previous laboratory experiment showed that Ca cations formed insoluble complexes with organic molecules whereas Na cations were mainly found in dissolved organic matter from soil suspensions [12]. Another experiment also showed that calcium bridging can result in reducing the losses of organic material upon photo-oxidation by approximately 7% [13]. Recently, a study showed that Ca bound to SOM fractions was the main component of soil organic carbon in two calcareous soils, highlighting the role of Ca cations rather than carbonate precipitation in stabilizations of soil organic carbon [14]. However, when inorganic carbonate exists as nanoparticles, its huge specific surface area can be important to binding SOM [15]. Calculations from a study showed that continuous incorporation of straw and manure into soils in the arid and semi-arid regions also enhanced carbonate accumulation in soil profiles[16]. Up to now, little has been understood regarding the interactions among Ca cations, carbonates and organic molecules in calcareous soil.

Under four fertilization treatments (Control, CK; chemical nitrogen, phosphorus and potassium, NPK; and NPK plus manure, NPKM; and manure, M from a long-term field experiment, combing with the promising FTIR-2DCOS and HRTEM techniques, the objectives of this paper were thus to investigate properties of soil and soil dissolved organic matter (DOM) by chemical analysis and fluorescence excitation emission matrix (EEM) spectroscopy and to observe the possible existence of nanominerals in soil DOM using high resolution-transmission electron microscopy (HR-TEM). In addition, we aimed to demonstrate the binding features of Ca^2+^ and DOM using a two-dimensional (2D) Fourier transform infrared (FTIR) spectrometer.

## Materials and Methods

### Soil sampling

The long-term field experiment has been established since 1990 at Urumqi (43°57′26″ N, 87°46′45″ E, 600 m in altitude), Xinjiang Province, China. This site represents an arid and semiarid desert region with gray desert soil or haplic calcisol according to FAO soil classification (pH 8.1 and SOC 8.8 g kg^-1^ in 1990). The authority responsible for the site is an experiment field site of Research Institute of Soil & Fertilizer and Agricultural Water Conservation in Urumqi. This soil originates from loess parent materials and experiences 299 mm of annual rainfall, 2570 mm of annual evaporation, an annual average temperature of 7.7°C, and 156 days without frost. Only one season of crops (wheat, maize or cotton) was grown each year, i.e., 14 seasons of wheat, 8 seasons of maize and one season of cotton (in 1999) were grown from 1990 to 2013. Four treatments from the long-term experiment have been selected for this study, i.e., CK, which did not contain any fertilizers; NPK, which contained 242 kg N ha^−1^, 60 kg P ha^−1^, and 50 kg K ha^−1^; NPKM, which contained 85 kg N ha^−^, 22 kg P ha^−1^, 10 kg K ha^−1^and 30 kg K ha^−1^ goat manure (10.1 g N kg^−1^, 2.2 g P kg^−1^ and 5.3 g K kg^−1^); and M, which contained only goat manure at 60 ton ha^-1^. 60% ofthe total nitrogen and 100% of the phosphate, potassium and manure were applied as the basic fertilizer before sowing, the remaining 40% nitrogen at the first irrigation in spring.These rates of fertilizers are applied for wheat, whereas those for maize and cotton are not listed here [11]. Winter wheat (Triticumaestivum) was sown in early September and harvested around 20 July in following year. Spring wheat was sown around 20 March and harvested around 25 July. Maize wassown around 25 April and harvested around 20 September. In accordance with the rotation systems, 7 maize and 13wheat crops were planted (except cotton planting in 1999).Furrow irrigation was used before 2008, and drip irrigation technology has been applied since 2008. Irrigation water volume range from 5 250 to 6 750 m^3^.

Surface soil (0–20 cm) samples were collected in May 2013. Each plot (468 m^2^) was evenly separated into three regions randomly sampled with 10 cores. The sample was placed into a re-sealed plastic bag and taken to the laboratory within 2 days. Soil samples were mixed thoroughly, air-dried and then stored for further chemical analysis.

### SOC, TN content and soil pH

SOC and TN content of soil under CK,NPK,NPKM and M were measured with a CN analyzer (VarioEL, Elementar GmbH, Hanau, Germany), and a soil to water ratio of 1:5 was used to measure pH.

### Extraction of DOM

Soil DOM was extracted with deionized water (1:5 w/v)on a horizontal shaker(170 rpm) for 24 h at 20°C and then centrifuged at 3000gfor 10 min. The supernatant suspension was passed through a 0.45-μm membrane filter and then stored for further study.

### Metals in DOM

An inductively coupled plasma-optical emission spectrometer (Perkin Elmer, Model Optima 7000 DV, USA) was used to quantify Al, Ca, Mg, Fe, Mg and Si in DOM. The ICP–OES operational conditions are represented in Table 1. All measurements were performed in triplicate.

**Table 1:** Operational conditions used for the determination of elemental impurities by ICP OES.

Parameter	ICP-OES
RF power, W	1400
Plasma gas flow rate, L min^−1^	15.0
Auxiliary gas flow rate, L min^−1^	1.2
Nebulizer gas flow rate, L min^−1^	1.15
Spray chamber	Cyclonic
Nebulizer	GenCone
Observation view	Axial
Analyte	Emission line, nm
Al	396.152 (I)
Ca	393.366 (II)
Fe	238.204 (I)
Mg	285.213 (I)
Si	181.972(I)

(I) atomic and

(II) ionic emission lines.

### Analysis of EEM spectroscopy of DOM

Before measurement, all DOM samples were further diluted with deionized water until the final concentration of dissolved organic carbon(DOC) was less than 10 mg L^−1^. The method described by Yu and colleagues 2012 was taken for the measurement and the analysis of EEM techniques Fluorescence EEM spectra of the DOM were measured using a Varian Cary Eclipse spectrofluorimeter.To obtain the EEM spectra, excitation wavelengths (Ex) were incrementally increased from200 to 400nmat 5-nm steps, whereas the emission varied from 200 to 500 nm in 10-nm increments.

### High resolution-transmission electron microscopy (HRTEM) analysis of DOM

Freeze-dried Samples of DOM were used to characterize the size, morphology and chemical composition using TEM. Freeze-dried samples of DOM were dissolved in alcohol and deposited on a carbon coated copper grid. HRTEM observation was performed on a a JEM2100F field emission gun TEM(JEOL, Japan). The HRTEM Selected area electron diffraction (SAED) measurements and energy dispersive X-ray(EDX)were made using the 200 keV electron beam to characterize the sample appearance.

### Titration of DOM with Ca^2+^

The Ca^2+^ titration was conducted in a brown sealed bottle that contained 30 ml DOM fraction solution with different CaCl_2_ concentrations (ranging from 0 to 100 mmol.L^-1^). All titration solutions were placed on a horizontal shaker for 24 h at 20°C for solution equilibrium.

### Analysis of FTIR spectroscopy and 2DCOS analysis

Freeze-dried samples of DOM-Ca^2+^ complexes from the above titrations were prepared. FTIR spectra were performed by adding 1 mg of DOM-Ca^2+^complex to100 mg of potassium bromide (KBr, IR grade). The FTIR spectra were collected by using a Nicolet 370 FTIR spectrometerover the range 4000–400 cm^-1^.Prior to 2D analysis, the FTIR spectra were normalized by summing the absorbance from 4000–400 cm^-1^and multiplying by 1000. Subsequently, normalized FTIR spectra were analyzed using principal component analysis (PCA) to reduce the level of noise.

In this study,the2D correlation spectra were obtained by following the methods of Noda and Ozaki[17,18] and finallyproduced by the 2Dshige software (Kwansei-Gakuin University, Japan). Considering that excitation bands of the organic substances(i.e., amide, carboxylic acid, and carbohydrate functional groups as well as silicon-oxygen linkage) were dominated in the region from 1800 cm^-1^ to 900 cm^-1^, we focus on this range for further study [19].

### Statistical analysis

SPSS ver.11 was used for these analyses. The means and standard deviations of the data were calculated and statistically examined using analysis of variance and a Duncan’s multiple range test. The significance level used for the mean separation was *P*<0.05.

## Results

### Soil pH, SOC and STN

Long-term fertilization by different fertilizer treatments has significantly (P <0.05) changed the soil pH, SOC and total soil N of surface (0–20 cm) soils (Table 2). In detail, the NPK and NPKM treatments significantly decreased the soil pH (P <0.05), whereas the M treatment maintained the soil pH. The addition of manure treatments (NPKM and M) significantly (P<0.05) increased the content of SOC and the soil total N, which were ranked by descending order as M>NPKM >NPK >CK. Additionally, M treatment increased SOC and STN by 1.86- and 2.52-times higher than CK, respectively.

**Table 2:** Soil pH, SOC and total soil N of surface soil samples in 2013 from different treatments of the long-term field experiment from Urumqi, Xinjiang, China.

Treatments	pH (H_2_O)	SOC /g kg^−1^	STN / g kg^−1^
CK	8.06 (0.05) a	8.95 (0.30) d	0.62(0.08) d
NPK	7.89 (0.02) b	12.02 (0.25) c	1.00 (0.06) c
NPKM	7.81 (0.06) b	14.77 (0.40) b	1.30(0.05) b
M	8.09 (0.03) a	16.50 (0.08) a	1.56(0.06) a

Data represent the means of three analytical replicates of the composite sample from each treatment with standard deviations in parentheses. Mean values in the same column followed by the same letter are not significantly different at P≤0.05 according to Duncan’s least significant difference (LSD) test.

### Metal concentrations of DOM

The concentrations of Ca, Fe, Mg, Si of DOM were significantly (P <0.05) altered by long-term application of different fertilizers (Table 3). Ca existed in all treatments and had significantly higher concentrations than that of other metals. In contrast, trivalent metals (Al and Fe) of DOM were detected at one to two orders of magnitude lower than Ca in all treatments. Application of organic manure significantly increased the concentrations of Ca, Mg and Si. However, the decreased concentrations of trivalent metals (Al and Fe) of DOM in all fertilizer treatments implied that trivalent metals occurred in low levels in the desert soil and were diluted by organic matter due to complex formation.

**Table 3:** Some element concentrations in DOM of surface soil samples in 2013 from different treatments of the long-term field experiment in Urumqi, Xinjiang, China.

Treatments	Al (mg.l^-1^)	Ca (mg.l^-1^)	Fe (mg.l^-1^)	Mg (mg.l^-1^)	Si (mg.l^-1^)
CK	1.3 (0.02) a	33.7 (1.12) d	0.8 (0.02) a	4.5 (0.12) d	23.2(1.01) b
NPK	0.5 (0.01) b	66.7 (0.50) c	0.1 (0.02) c	9.2 (0.06) c	22.2 (1.12) b
NPKM	0.5 (0.02) b	75.6 (0.65) a	0.1 (0.02) c	15.2 (0.08) b	29.4 (0.98) a
M	0.5 (0.01) b	69.6 (0.81) b	0.2 (0.01) b	16.3 (0.08) a	30.2 (1.05) a

Data represent the means of three replicates with S.D. in parentheses. Mean values in the same column followed by the same letter are not significantly different at P≤0.05 according to Duncan’s least significant difference (LSD) test.

### EEM of DOM

**Fig 1:**
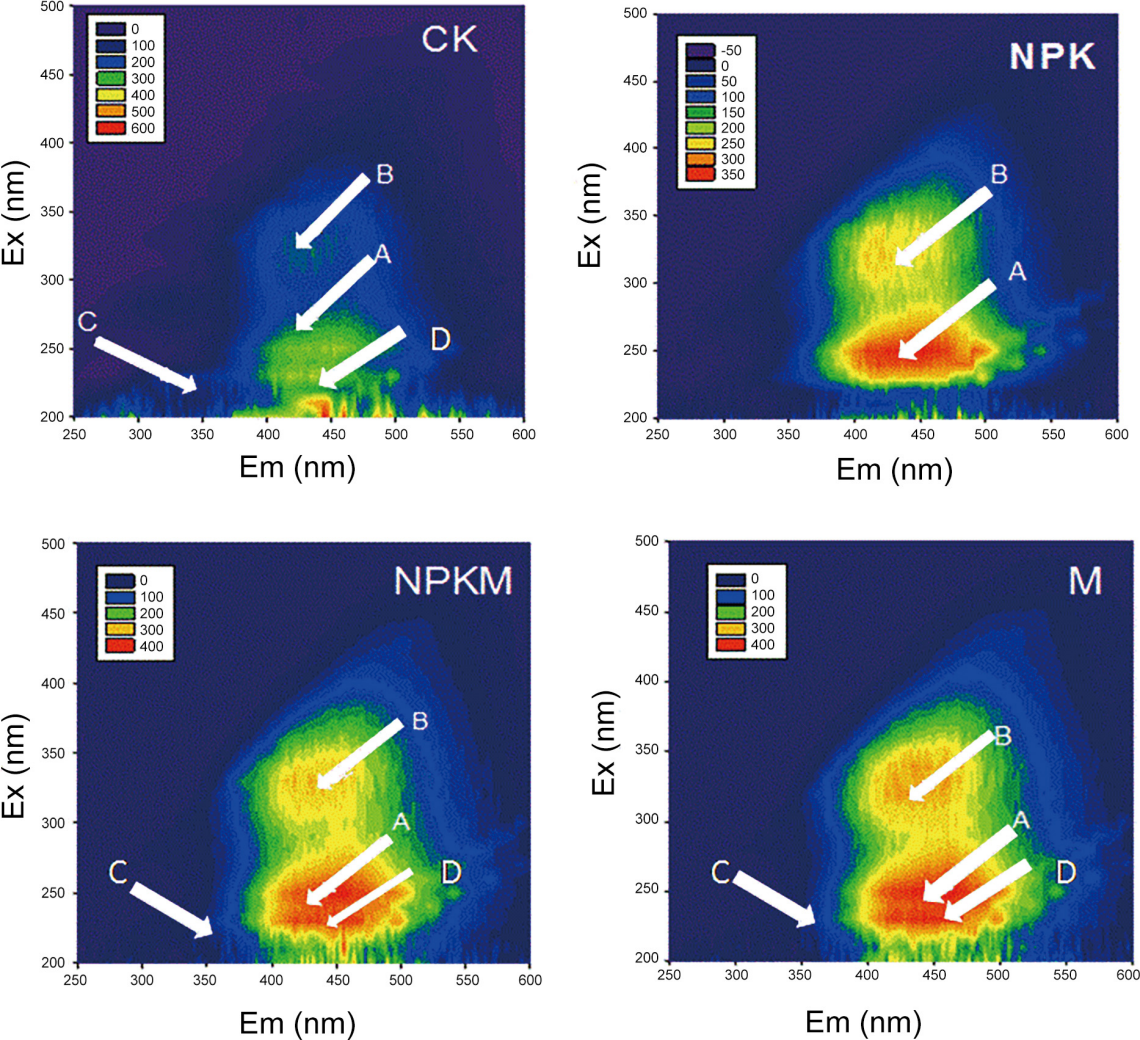
Fluorescence excitation-emission matrix (EEM) spectra of dissolved organic matter (DOM) for soils from the CK, NPK, NPKM and M treatments, respectively, in the long term experiment. Specifically, Peaks A(Ex/Em of 240/420), B(Ex/Em of 320/420), C(Ex/Em of 280/350) and D(Ex/Em of 230/420)referred to fulvic acid-like (peaks A and D), humic acid-like (peak B) and protein-like (peak C) substances, respectively.

Peaks in the fluorescence EEM spectra of DOM samples from the four treatments are shown in Fig 1. Peaks A(Ex/Em of 240/420), B(Ex/Em of 320/420), C(Ex/Em of 280/350) and D(Ex/Em of 230/420)referred to fulvic acid-like (peaks A and D), humic acid-like (peak B) and protein-like (peak C) substances, respectively[20]. For CK, soil DOM was occupied by fulvic acid-like (peaks A and D) substances with small quantities of humic acid-like (peak B) and protein-like (peak C) substances for the fertilizer-amended treatments NPK, NPKM and M, signals of the humic acid-like substances increased and those of protein-like disappeared although fulvic acid-like substances dominated the DOM (Peaks A and D). The weal of Ex/Em 230/420 signal (Peak D) in the NPK treatment sample suggested that application of chemical fertilizers (NPK treatment) simplified the DOM fluorescence spectra.

### FTIR spectra

**Fig 2:**
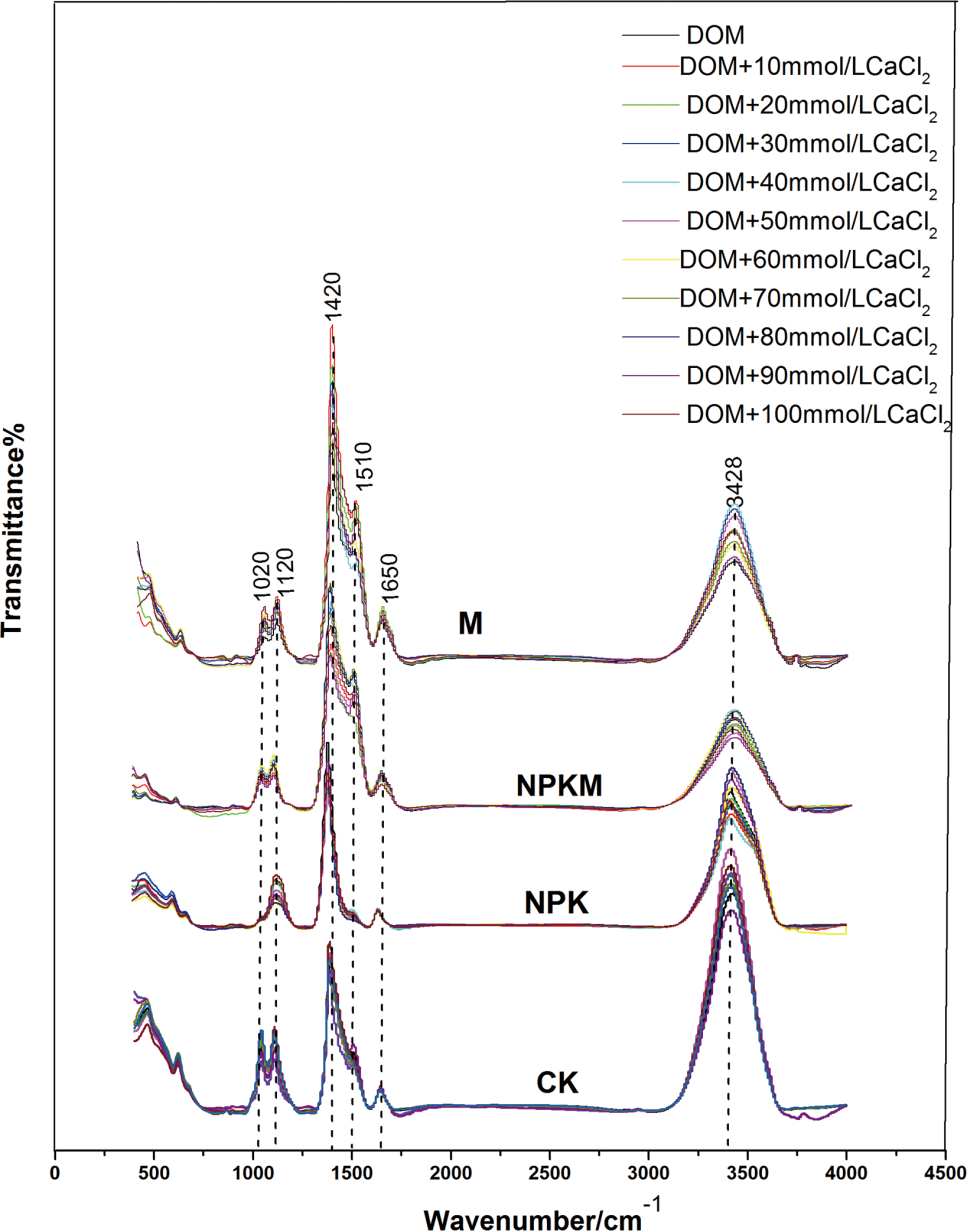
FTIR spectra of the titration of the CK, NPK, NPKM and M treatment depending on the concentrations of CaCl_2_changes(from 0mmol/L to 100mmol/L).

The FTIR spectra of DOM-Ca^2+^ complexes from titration with Ca^2+^ of the DOM samples from the studied treatments are shown in Fig 2. The peaks of various spectra strongly overlapped. The spectra from the NPK treatment were different from these of the CK, NPKM and M treatments. The aliphatic O–H and C–OH stretch (1120 cm^-1^), carboxylic O-H stretch (1420 cm^-1^), amide C = O stretch (amide I) (1650 cm^-1^) and hydrogen bonded O–H stretch (3428 cm^-1^) could be detected in all treatments. However, the Si–O (1080 cm^-1^) stretching of silicate and amide N–H bending (amide II) (1510–1520 cm^-1^) disappeared in the NPK treatment samples, indicating that NPK treatment weakened the possible association of DOM with silicon nanoparticles and DOM with metals that form complexes with nitrogen atoms and amide of the II group by its loneelectron pair.

### FTIR synchronous maps

**Fig 3:**
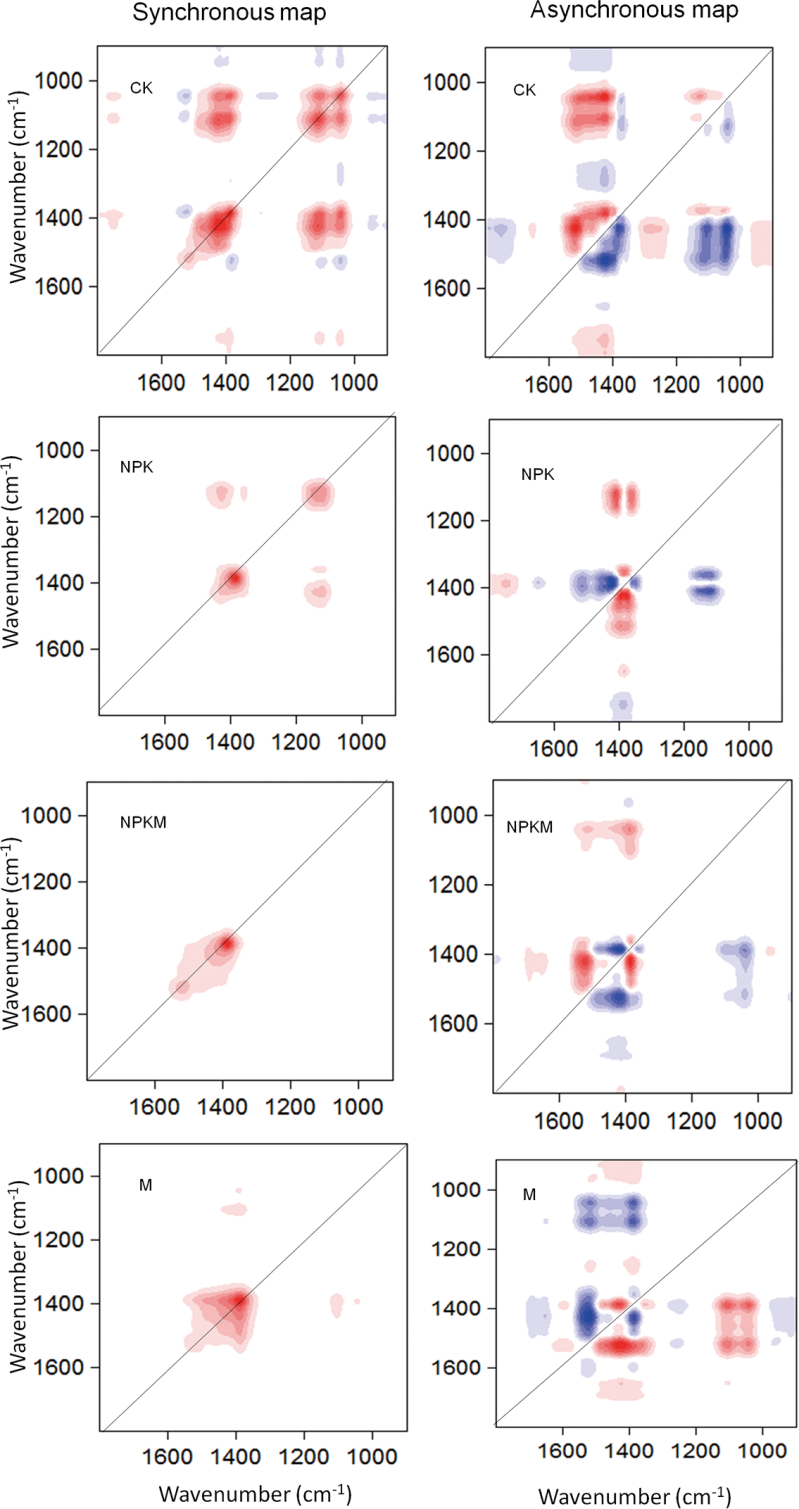
Synchronous and asynchronous 2D correlation maps generated from the 1800–900 cm^-1^ region of the FTIR spectra of dissolved organic matter in the CK, NPK, and NPKM treatments over Ca^2+^. Red represents positive correlation, and blue represents negative correlation; a higher color intensity indicates a stronger positive or negative correlation.

The results of 2D-FTIR are listed in Table 4. The synchronous maps collected from FTIR spectra of DOM with increased Ca^2+^ are illustrated in Fig 3. The orders of the intensity of auto-peaks reflect the peaks with great change under different Ca^2+^ concentration [21,22], were arranged as follows: 1420 cm^-1^> 1380 cm^-1^> 1120 cm^-1^> 1080 cm^-1^> 1510 cm^-1^ for CK, 1380 cm^-1^> 1120 cm^-1^ for NPK treatment, and 1380 cm^-1^> 1510 cm^-1^ for NPKM and M treatment, indicating that application of chemical fertilizers (NPK treatment) weakened the role of amides II (1510 cm^-1^) whereas application of manure (NPKM and M treatments) strengthened the role of organic functional groups (1380 cm^-1^ and 1510 cm^-1^) in complex formation with Ca^2+^. However, when no fertilizer was applied, several groups responded to the Ca^2+^ addition.

**Table 4:** Synchronous and asynchronous 2D correlation results.

Treatments	Synchronous results		Asynchronous results sequences of the intensity changes
	orders of the intensity of auto-peaks	positive correlations	
CK	1420 cm^-1^> 1380 cm^-1^> 1120 cm^-1^> 1080 cm^-1^> 1510 cm^-1^	1420 with 1120 cm^-1^,1420 with 1120 cm^-1^ 1380with 1120 cm^-1^,1380 with 1080 cm^-1^ 1120 with 1080 cm^-1^	1510> 1420 >1120> 1080 > 1380
NPK	1380 cm^-1^> 1120 cm^-1^	1420 with 1120 cm^-1^,1380 with 1120 cm^-1^	1380> 1420(1510 1120)
NPKM	1380 cm^-1^> 1510 cm^-1^	1380 with 1120 cm^-1^, 1380 with 1080 cm^-1^	1510(1380)>1420>(1120,1080)
M	1380 cm^-1^> 1510 cm^-1^	1380 with 1120 cm^-1^, 1380with 1080 cm^-1^	1420>(1120,1080)>1380(1510)

Synchronous positive correlations revealed that the intensity variations of crosspeaks proceed in the same direction upon perturbation, whereas negative crosspeaks show changes in opposite direction. In the present study, positive correlations occurred at 1420 cm^-1^ with 1120 cm^-1^, 1420 cm^-1^ with 1120 cm^-1^, 1380 cm^-1^ with 1120 cm^-1^, 1380 cm^-1^ with 1080 cm^-1^ and 1120 cm^-1^ with 1080 cm^-1^ for CK; at 1420 cm^-1^ with 1120 cm^-1^, and 1380 cm^-1^ with 1120 cm^-1^ for NPK treatment; and at 1380 cm^-1^ with 1120 cm^-1^, and 1380 cm^-1^ with 1080 cm^-1^ for M treatment. Other weak synchronous cross-peaks existed in the DOM samples from CK but did not exist in samples from fertilized treatments.

Co-variation of organic functional groups and Si-O linkage suggested that organic matter and Si-O-containing nanoparticles were involved in binding the Ca^2+^ in DOM from the CK and M treatments. Obviously, bands at 1510 cm^-1^ and 1080 cm^-1^ hardly occurred in NPK treatment.

### FTIR asynchronous maps

The asynchronous map which is anti-symmetric over the diagonal line without auto-peaks indicates the order with which different functional groups bind to Ca^2+^[21, 22]. From the asynchronous map in Fig 3, the peak at 1510 cm^-1^ is positive with the peaks at 1420 cm^-1^, 1120 cm^-1^ and 1080 cm^-1^ for CK and NPKM treatments but is negative with the peak at 1380 cm^-1^ for NPK and with the peaks at 1420 cm^-1^, 1120 cm^-1^ and 1080 cm^-1^ for M treatments; the peak at 1420 cm^-1^ is positive with the peaks at 1380 cm^-1^, 1120 cm^-1^ and 1080 cm^-1^ for CK treatment and with the peak at 1380 cm^-1^ for M treatment and the peak at 1120 cm^-1^ for NPK treatment. However, this peak is negative with the peaks at 1380 cm^-1^ for NPK and NPKM treatments. The peak at 1380 cm^-1^ is negative with the peaks at 1120 cm^-1^ and 1080 cm^-1^ for CK and M treatments but is positive with the peaks at 1120 cm^-1^ and 1080 cm^-1^ for NPKM treatment and the peak at 1120 cm^-1^ for NPK treatment. The correlation between peaks at 1120 cm^-1^ and 1080 cm^-1^ is shown from only the CK samples with addition of Ca^2+^ and is positive. Other weak asynchronous cross-peaks existed in the DOM samples from CK treatment but do not exist in samples from fertilized treatments.

The sequences of the intensity changes under different Ca^2+^ concentrations can be obtained by the cross-peaks of the 2D correlation spectra [21–23]. According to Noda’s rule, after addition of Ca^2+^, 1510 cm^-1^ changed before 1420 cm^-1^, 1120 cm^-1^ and 1080 cm^-1^ for CK and NPKM treatments but after 1380 cm^-1^ for NPK and after 1420 cm^-1^, 1120 cm^-1^ and 1080 cm^-1^ for M treatments; 1420 cm^-1^ changed before 1380 cm^-1^, 1120 cm^-1^ and 1080 cm^-1^ for CK and before 1380 cm^-1^ for M treatment and before 1120 cm^-1^ for NPK treatment but after 1380 cm^-1^ for NPK and NPKM treatments; 1380 cm^-1^ changed after 1120 cm^-1^ and 1080 cm^-1^ for CK and M treatments but before 1120 cm^-1^ and 1080 cm^-1^ for NPKM treatment and before 1120 cm^-1^ for NPK treatment; and the 1120 cm^-1^ peak changed before 1080 cm^-1^ for CK.

Because the change of intensity of 1080 cm^-1^ (Si-O) occurs prior to the changes in intensities of organic functional groups (1510 cm^-1^ and 1380 cm^-1^) in DOM for M treatment with increasing Ca^2+^ concentration, it is suggested that Si-O nanoparticles have higher affinity with Ca^2+^ than do organic functional groups.

### HRTEM images

**Fig 4:**
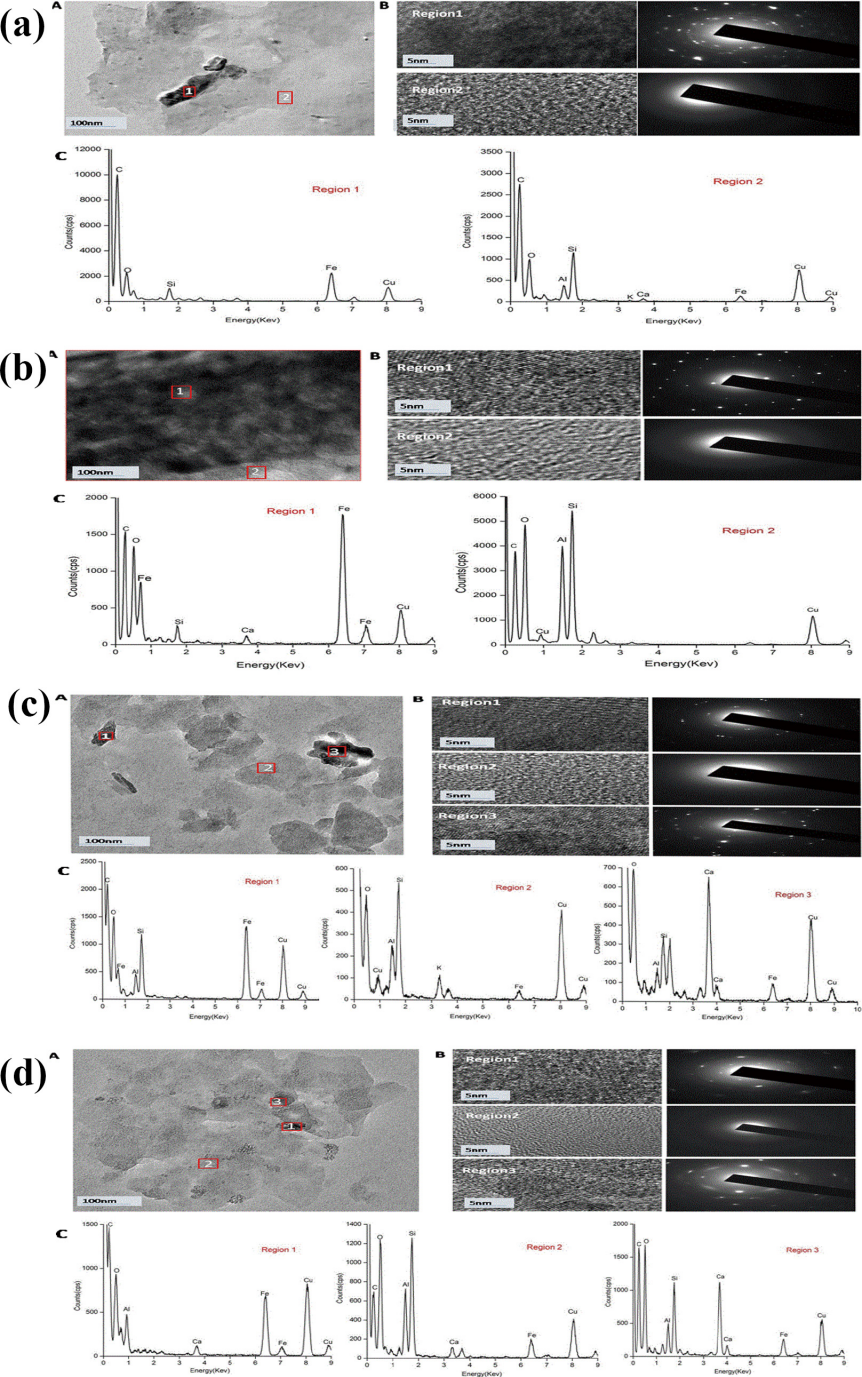
High resolution transmission electron microscopy (HRTEM) images of soil dissolved organic matter from CK(a), NPK(b), NPKM(c) and M(d). A:TEM images; B:HRTEM images and selected area electron diffraction pattern of the three regions (black region is completely crystalline, whereas the gray region remains amorphous); C:EDS images.

To further confirm the formation of nanominerals in the soil under different types of fertilization, TEM images were applied to investigate the morphology, diffraction pattern and elemental maps of soil nanominerals. The HRTEM spectra showed nanoparticles existed as the major mineral phases. The TEM images of DOM from NPKM (Fig 4C) and M (Fig 4D) treatments had three distinct types of nanoparticles (i.e., region 1, region 2 and region 3) whereas these from the CK (Fig 4A) and NPK (Fig 4B) treatments had two (i.e., region 1 and region 2). The nanoparticles showed different size, shape and chemical composition. The electron diffraction patterns showed that nanoparticles in regions 1 and 3 were crystalline minerals, whereas those in region 2 were amorphous or short-range-order minerals. EDS analysis showed that crystalline nanoparticles in region 1 were dominated by Fe, Si/Al and O, whereas those in region 3 were dominated by Ca, Si and O. In contrast, amorphous nanoparticles in region 2 were dominated by Si, Al and O. The HRTEM observation and metal analysis (as seen in Table 2) suggested that crystalline nanoparticles of Ca carbonate and amorphous nanoparticles of Si compounds possibly played significant roles in preservation of SOM in terms of Ca and Si richness in the DOM of NPKM and M treatments compared with nanoparticles of Fe and Al compounds due to decreased concentrations of Fe and Al in DOM samples.

## Discussion

### Impacts of long-term application of different fertilizers on soil properties

The results show that long-term agricultural fertilizer treatments have significant effects on the properties of the desert soil. The higher values of SOC and STN in the NPKM and M treatments compared with the control and NPK treatment confirm the importance of the fertilizer regime in altering soil properties. No matter whether chemical fertilizers was applied, long-term addition of manure significantly increased SOC and STN. Previous studies had confirmed the results[11, 24, 25]. The increase in surface SOC on arid cropland would be a result of intensive cropping through irrigation and fertilization that leads to enhanced plant growth and subsequent increased organic carbon input into the topsoil[16]. Moreover, long-term fertilization has significant effects on metal concentrations (Ca, Fe, Mg, Si, Al) of DOM in the desert soil. Ca and Mg existed in all treatments and had significantly higher concentrations than did other metals. By contrast, trivalent metals (Al and Fe) of DOM were detected at one to two orders of magnitude lower than Ca in all treatments. After long-term fertilization, the NPKM and M treatments has much higher values of Ca and Mg than those of the CK and NPK, which might have resulted from fertilization of calcium super-phosphate irrigation with Ca-rich waters[26] and/ or chemical weathering of calcium and magnesium silicate minerals[16].

Observations from the EEM results revealed that long-term fertilization by different fertilizers had little effect on the fluorescent substances, which was confirmed by a previous study by Wu Jun et al.,[27]. The little effect of long-term fertilization by different fertilizers on the fluorescent substances revealed that the interaction organisms with soil mineral played a key role in affecting the SOM, while the application of manure showed minor effect [27].

### Effects of fertilization on the binding characteristics of organic ligands to Ca^2+^

The FTIR spectroscopy was a useful method to observe the chemical properties of different substances [21]. In this paper, the spectra of the NPK treatment were quiet different from those of the CK, NPKM and M treatments. The main IR absorption bands of the different treatments strongly overlapped. Meanwhile, the results revealed that the intra-hydrogen bond (> 3400 cm^-1^) which can be found in all treatments played an important role in binding with Ca^2+^ (Fig 1). IR bands between 3600 and 3200 cm^-1^ are used to study the hydrogen bond network in nanominerals, which is a critical component of soil nanominerals[28]. However, the Si–O stretching of silicate appeared only in the CK, NPKM and M treatments. In addition to the H-bond network (i.e., 3428 cm^-1^), it is reasonable to surmise that the nanoparticles of Ca carbonate and amorphous nanoparticles of Si compounds possibly formed in soil DOM from the CK, NPKM and M treatments[22]. It is easy to assume that the addition of organic matter contributes to the formation of calcium nanominerals. A previous study showed thatthe formation of nanominerals weakened the bioavailability of organic matter [29]. This is consistent with the present results in that the long-term application of manure (NPKM or M treatment) significantly increased the SOC, total N and soil pH compared with that in the NPK at 0–20 cm. Therefore, the sequestration of SOM in the NPKM and M treatments resulted in the formation of calcium nanominerals in soil DOM, which could finally increase the storage of carbon by reducing the reactivity and bioavailability of organic matter.

The 2D-FTIR can determine the binding characteristics of SOM with Ca^2+^ at a more detailed level of functional organic groups than at the component level and present the relative binding affinities sequencing of different organic substances with Ca^2+^. Despite the minor effect of long-term application by different fertilizers on soils, 2D-FTIR analysis showed a mark effect on the binding of soil DOM with Ca^2+^. Observations from the 2DCOS FTIR spectroscopy showed that fertilization modified the binding characteristics of organic ligands to Ca^2+^ in soil DOM. First, in the 1800–900 cm^-1^range much more organic binds were bound to Ca^2+^ in the CK compared with that in the NPK, NPKM and M treatments. Second, the application of chemical fertilizers (NPK treatment) weakened the role of amides II (1510 cm^-1^), which finally induced the disappearance of protein-like substances in the EEM results. Compared with NPK, the Si-O content (1020cm^-1^) in the CK, NPKM and M was more important for the binding to Ca^2+^.According to the Synchronous Maps, the co-variation of organic functional groups and Si-O linkage suggested that organic matter and Si-O-containing nanoparticles were involved in binding Ca^2+^ in DOM from CK and M treatments. However, the *asynchronous Maps* suggested that Si-O nanoparticles have higher affinity with Ca^2+^ than do organic functional groups in the NPK and NPKM treatments. The results indicated the high possibilities of the existence of Ca-Si-O nanoparticles in the NPKM and M treatments, which was later confirmed by the HRTEM results.

### Effects of fertilization on the nanominerals in gray desert soil-the formation of calcium nanocrystalline

The binding of organic ligands to Ca^2+^ in soil DOM could affect the type of soil nanominerals. In this study, we found that the formation of calcium nanocrystalline phases occurred under NPKM and M treatment after long-term fertilization. Previous studies showed that rate of crystallization, and the Mg and Na concentration of the precipitating waters performing as indicators of environment were the key factor in affecting the morphology of calcium carbonate crystals. In Mg-rich sites, such as beaches or marine bottoms, micritic or fibrous aragonite and magnesian calcite cements form [30]. In combination with the results of the metal concentration (Table 2), the concentrations of Mg are much higher than Al and Fe, which will finally improve the growth of calcite. Combined with the results of the metal concentration, the formation of calcium nanocrystalline complexes in the binding of DOM with Ca^2+^ as an effective way to protect DOM, which finally contributed to the higher level of calcium and TOC in the NPKM and M treatments than in the CK and NPK treatments.

Results from the HRTEM images also revealed that the NPKM and M treatments had more amorphous nanominerals, whereas the NPK treatment had more crystalline nanominerals. The organic ligands in soil DOM, such as–OH and Si-O, could form amorphous nanominerals by network of nanominerals, which finally prevents the formation of sheets of crystalline minerals. EDS images revealed that the NPK was dominated by crystalline nanominerals (i.e., ferrihydrite), composed of Fe and O, while amorphous nanominerals (allophone or imogolite), composed of Al, Si, and O, were the main nanominerals in the other three treatments. The formation of non-crystalline minerals (i.e., allophone and imogolite) could significantly weaken the reactivity and bioavailability of organic matter [25]. Furthermore soil organic carbon storage and turnover, could controlled by the non-crystalline minerals (i.e., allophone, imogolite) because of that SOC concentrations positively correlate with allophone and imogolite. Long-term fertilization with the application of organic matter could affect the bioavailability and turnover of SOC by forming the non-crystalline minerals (i.e., allophone and imogolite). This is consistent with results obtained from long-term experiments elsewhere[30, 31].However, previous studies have been mainly concerned with the cropsoil in paddy soils. This study is the first to report the formation of calcium nanocrystalline in gray desert soil.

In conclusion, long-term organic applications (NPKM or M treatment) resulted in significant increases in organic matter contents (SOC, total N) of soils located within semi-arid climatic conditions. Meanwhile, long term fertilization can modify the binding characteristic of DOM to Ca^2+^ in gray desert soil. Among organic ligands in soil DOM, CH deformations in aliphatic groups and Si–O stretching of silicate plays an important role in binding to Ca^2+^. Moreover, long term fertilization can affect the morphology and diffraction of nanominerals in soil DOM. Dominant crystalline nanominerals were in NPK, whereas amorphous minerals occurred under the CK, NPKM and M treatments. Meanwhile, the formation of calcium nanocrystalline and non-crystalline Fe in NPKM and M treatments was an effective way to protect the DOM in gray desert soil. In summary, a further study of the binding capability of Ca2^+^ with DOM contributes to a better understanding of soil C sequestration and global C cycling in gray desert soil.

## Abbreviations

DOM: dissolved organic matter
SOM: soil organic matter
STN: soil total nitrogen
